# Epidemiology of childhood tuberculosis and predictors of death among children on tuberculosis treatment in central Ethiopia: an extended Cox model challenged survival analysis

**DOI:** 10.1186/s12889-023-16183-9

**Published:** 2023-07-04

**Authors:** Abay Burusie, Fikre Enquesilassie, Nicole Salazar-Austin, Adamu Addissie

**Affiliations:** 1 Department of Public Health, College of Health Sciences, Arsi University, Asella, Ethiopia; 2 School of Public Health, College of Health Sciences, Addis Ababa University, Addis Ababa, Ethiopia; 3 Division of Pediatric Infectious Diseases, Johns Hopkins University School of Medicine, Baltimore, Maryland USA

**Keywords:** Epidemiology, Survival, Childhood tuberculosis

## Abstract

**Background:**

Childhood tuberculosis (TB) was poorly studied in Ethiopia. This study aimed to describe the epidemiology of childhood TB and identify predictors of death among children on TB treatment.

**Methods:**

This is a retrospective cohort study of children aged 16 and younger who were treated for TB between 2014 and 2022. Data were extracted from TB registers of 32 healthcare facilities in central Ethiopia. Phone interview was also conducted to measure variables without a space and not recorded in the registers. Frequency tables and a graph were used to describe the epidemiology of childhood TB. To perform survival analysis, we used a Cox proportional hazards model, which was then challenged with an extended Cox model.

**Results:**

We enrolled 640 children with TB, 80 (12.5%) of whom were under the age of two. Five hundred and fifty-seven (87.0%) of the enrolled children had not had known household TB contact. Thirty-six (5.6%) children died while being treated for TB. Nine (25%) of those who died were under the age of two. HIV infection (aHR = 4.2; 95% CI = 1.9–9.3), under nutrition (aHR = 4.2; 95% CI = 2.2-10.48), being under 10 years old (aHR = 4.1; 95% CI = 1.7–9.7), and relapsed TB (aHR = 3.7; 95% CI = 1.1–13.1) were all independent predictors of death. Children who were found to be still undernourished two months after starting TB treatment also had a higher risk of death (aHR = 5.64, 95% CI = 2.42–13.14) than normally nourished children.

**Conclusions:**

The majority of children had no known pulmonary TB household contact implying that they contracted TB from the community. The death rate among children on TB treatment was unacceptably high, with children under the age of two being disproportionately impacted. HIV infection, baseline as well as persistent under nutrition, age < 10 years, and relapsed TB all increased the risk of death in children undergoing TB treatment.

## Introduction

Childhood tuberculosis (TB) was first included in the World Health Organization (WHO) global report in 2012, with an estimated 0.5 million new TB patients under the age of 15, accounting for 5.7% of all TB patients in 2011 [1]. WHO estimates 1.2 million children developed TB disease in 2021, which accounts for 11% of the total TB burden [2].

WHO estimated children accounted for 4.6% and 14% of TB-related mortality globally in 2011 and in 2021, respectively, but only 5.7% and 11% of TB incidences in 2011 and 2021, respectively [1, 2]. Modeling studies suggest greater than 96% of child TB deaths occur in children not receiving treatment [3].

Ethiopia has long been one of the 22 [1] or 30 countries with a high TB burden  [2, 4], ranking seventh in the world in 2021 [2]. In Ethiopia, WHO estimates that about 20,000 children under the age of 15 (i.e. 11.6% of all TB patients) become ill with TB, accounting for 9.5–14.9% of all TB patients in 2017 [4].

GeneXpert MTB/RIF® has been used as the primary tool for TB diagnosis in Ethiopia since 2015 [5]. Xpert is a more sensitive and specific test than microscopy, allowing for more accurate data generation to describe childhood TB epidemiology [6–8].

Ramos et al. discovered that 4.1% of children on TB treatment died in a rural hospital in southern Ethiopia between 1998 and 2015. However, the study did not identify predictors of death [9]. Death rates from diagnosed and treated childhood TB have been reported as less than 1% [10, 11]. To reduce the death rate among children receiving TB treatment to the greatest extent possible, we must identify the predictors of death to implement risk factor-focused interventions in addition to anti-TB treatment [12–14]. Although it has been shown in other parts of the world that the Bacillus Calmette Guerin (BCG) vaccine is effective at preventing TB deaths in children [15] and under nutrition increases the risk of death from TB in adults [16, 17], studies in Africa and Ethiopia [11, 18–27] left these factors out. The aim of this study was to describe the epidemiology of childhood TB and identify predictors of death in children with presumed drug-susceptible TB in central Ethiopia between 2014 and 2022.

## Methods

### Study site and period

This study was carried out in TB treatment facilities in Addis Ababa city, Adama, and Bishoftu towns. The study included data from children who were treated for TB disease between June 6, 2014, and February 16, 2022.

### Study design and population

We used a retrospective cohort study design. According to Ethiopian national guidelines [28], new or relapsed pulmonary TB (PTB) patients and all forms of extra-pulmonary TB (EPTB), except TB meningitis and osteoarticular TB, are treated for at least 6 months. The intensive phase lasts two months and is treated with a combination of rifampicin, isoniazid, pyrazinamide, and ethambutol. The continuation treatment, which lasts four months, is treated with rifampicin and isoniazid. TB meningitis and osteoarticular TB are treated for a total of 12 months, including two months of the initiation phase and 10 months of the continuation phase, with the same drugs as described above.

This study included children aged 16 years and younger who had been diagnosed with PTB or EPTB, either for the first time treatment or as a relapse or retreatment, with presumed drug-susceptible TB.

Children who were transferred to the study healthcare facilities after beginning TB treatment elsewhere were excluded from the study because baseline data, such as nutritional status at the start of treatment, were unable to be obtained. Children who had switched from TB to another diagnosis despite having completed TB treatment were also excluded.

### Study variables

The time-to-death (in months) of a child during TB treatment was our outcome variable. The end of follow-up periods for the 6 and 12 month regimens were 6 and 12 months, respectively.

The independent variables of this study included child’s BCG vaccination status at birth or within 15 days, age, sex, HIV status, TB treatment history, TB type, nutritional status at time of TB treatment start, and nutritional status two months after TB treatment initiation.

From TB registers, we obtained patients’ weight, height (length for children under the age of two), and mid-upper-arm circumference (MUAC). Body mass index (BMI)-for-age-z-score, weight-for-height/length-(WH/L)-z-score, MUAC, or weight-for-age percentile indices were used to determine the nutritional status of the children based on their appropriateness for different age groups and availability. The cutoffs for the indices for normal, moderate acute malnutrition (MAM), and severe acute malnutrition (SAM) are shown in Table 1. We used the weight for age chart developed by Centers for Disease Control and Prevention (CDC) for children aged 2 to 20 years to identify underweight when there was no height/length or MUAC measurement.

**Table 1:** Indices used to assess the nutritional status of childhood TB patients (≤16 years old) in central Ethiopia, 2014 to 2022

Option	Child age	Index	Cutoffs for nutritional status		
			Normal	MAM	SAM
Option 1 [29]	5–18 years	BMI-for-age-z-score	-2 ≤ z ≤ + 1	-3 ≤ z < -2	z < -3
Option 2 [30]	0–59 months	WH/L-z-score	-2 ≤ z ≤ + 1	-3 ≤ z < -2	z < -3
Option 3 [29]	6–59 months	MUAC	≥ 125 mm	115–125 mm	< 115 mm
	5–9 years	MUAC	≥ 145 mm	135–145 mm	< 135 mm
	10–14 years	MUAC	≥ 185 mm	160–185 mm	< 160 mm
Option 4 [31]	2–20 years	CDC weight-for-age percentile	5th -95th	<5th is underweight	

### Sample size determination

We used the sample size determination formula for the Cox proportional hazards model that compares the survival curves of two groups [32]. The total sample size was computed to be 628 patients with childhood TB (502 BCG-vaccinated versus 126 not vaccinated). The assumptions were a significance level of 5% and a power of 80% for a normal distribution [33], a postulated hazard ratio of 0.5 for death among children vaccinated with BCG compared to those unvaccinated, the probability of death in the unvaccinated and vaccinated groups equaling 0.08 and 0.24, respectively [34], and the ratio of vaccinated to unvaccinated equaling four based on evidence that 81% of the population had BCG scars and 19% did not [35].

### Sampling technique

Facilities were randomly chosen, stratifying the Addis Ababa, Adama and Bishoftu areas. From Addis Ababa, three hospitals were chosen at random from a total of six governmental hospitals, and 23 governmental health centers were chosen at random from a total of 95 centers that provide TB treatment services. The government hospital and two health centers in Adama were chosen at random from among five that offer TB services. One of Bishoftu’s two hospitals and two of its four health centers were also chosen at random. As a result, the study included 32 healthcare facilities, and all children treated for tuberculosis in those facilities who met the inclusion criteria were studied.

### Data collection process

The TB register’s content was used to generate a checklist and extract data. A structured questionnaire was administered via phone interviews to measure variables without a space and not recorded in the registers. Trained clinical nurses and health officers extracted the data and conducted the questionnaire. The principal investigator quality checked 100% of the data for accuracy and completeness.

### Data analysis

EpiData version 3.1 was used to enter data, which was then exported to Stata version 14 for cleaning and analysis. Data were described using frequency tables and a line graph. The log-log graphical proportional hazards (PH) assumption test was first performed and independent variables that demonstrated parallel curves for their categories were considered to be included in the Cox PH survival analysis model. To supplement the graphical assumption test, we ran a statistical goodness-of-fit test and variables with p-values greater than 0.05 were assumed to satisfy the PH assumption and be included in the Cox PH model. We conducted a disaggregated analysis on variables with crossing log-log curves in their respective categories, examining the survival function separately for survival times below and above the crossing point. In the final model, we challenged the Cox PH model with the extended Cox model for variables that were ambiguous with the graphical assumption test but satisfied the goodness-of-fit test. A single covariate Cox PH model was run first to identify variables with p-values less than or equal to 0.20 for crude hazard ratio (HR) as candidates for the final multivariable Cox PH model. The log likelihood ratio test was used to select the best-fit model from the Cox PH and extended Cox models. Finally, covariates with p-values less than 0.05 for the adjusted hazard ratio (aHR) were reported as independent predictors of death in children on TB treatment. The 95% confidence intervals (CI) for crude HR and aHR were also shown.

### Ethical consideration

 The study was approved by the Institutional Review Board of Addis Ababa University’s College of Health Sciences (protocol number: 057/19/SPH). All procedures followed were in accordance with the Helsinki Declaration. Before taking part in the study, children’s parents or guardians provided informed consent and older children provided informed consent to participate in the phone interview.

## Results

### Socio-demographic characteristics of childhood TB patients

A total of 650 children aged 16 years and younger who were treated for TB were identified from the TB registers. A phone interview was successful with 534 (83.4%) of the children’s families or guardians. During the phone interview, the caregivers or guardians of 10 (1.9%) of the children reported that further investigation revealed their children’s illnesses were lymphoma, brain stem glioma, and Crohn’s disease rather than TB. As a result, 640 children were included in this analysis: 524 (81.9%) children who could be reached by phone and 116 (18.1%) children who could not be reached by phone.

Of the 640 children, 368 (57.5%) were female. The mean (standard deviation) and median (inter-quartile range) ages of the patients were, respectively, 10.0 (5.4) and 12.0 (5.0 to 15.0) years. Four hundred and fifty-five children (71.1%) were Addis Ababa residents. Among the 519 children with a known daytime spending place, 376 (72.4%) were in school (including kindergarten), and 20 (3.9%) were in daycare, together accounting for 76.3% of the total TB-sick children involved in the study. But 123 (23.7%), were spending at home when they were diagnosed with TB of which 111 (90.2%) did not go to school (kindergarten) because they were not of school age (< 4 years).

One hundred and ninety-eight (52.7%) of those enrolled in school were in grades one through six. Two hundred and fifty-two (67.0%) were attending a public school at time TB diagnosis. One hundred and sixty-five mothers (32.6%) and 98 fathers (20.1%) of the children had no formal education. Of the total number of children, 560 (87.5%) were diagnosed with and treated for TB after 2015, when GeneXpert MTB/RIF® was introduced (Table 2).

**Table 2:** Socio-demographic characteristics of childhood TB patients (≤16 years old) and their families in central Ethiopia, 2014–2022

Characteristics	Category	Frequency (%)
Sex (*n* = 640)	Male	272 (42.5)
	Female	368 (57.5)
Age summary in years (*n* = 640)	Minimum = 0.2; Maximum = 16; Mean (standard deviation) = 10.0 (5.4); Median (IQR) = 12 (5 to 15)	
Region (*n* = 640)	Addis Ababa	455 (71.1)
	Oromia	165 (25.8)
	SNNPR	9 (1.4)
	Amhara	8 (1.3)
	Other	3 (0.5)
Where child was spending at the time of TB diagnosis (*n* = 519)	In school (including kindergarten)	376 (72.4)
	At home	123 (23.7)
	At daycare	20 (3.9)
Child’s education (*n* = 376)	Kindergarten	40 (10.6)
	Grade 1 to 6	198 (52.7)
	Grade 7 to 8	81 (21.5)
	Grade 9 to 10	57 (15.2)
The type of school the child was attending (*n* = 376)	Public	252 (67.0)
	Private	124 (33.0)
Mother’s education (*n* = 506)	Didn’t attend formal school	165 (32.6)
	Grade 1 to 6	66 (13.0)
	Grade 7 to 8	50 (9.9)
	Grade 9 to 12	115 (22.7)
	Diploma	31 (6.1)
	Degree and above	35 (6.9)
	Mother was not alive	44 (8.7)
Father’s education (*n* = 487)	Didn’t attend formal school	98 (20.1)
	Grade 1 to 6	57 (11.7)
	Grade 7 to 8	50 (10.3)
	Grade 9 to 12	129 (26.5)
	Diploma	40 (8.2)
	Degree and above	62 (12.7)
	Father was not alive	51(10.5)
Year the child diagnosed with TB (*n* = 640)	2014	13 (2.0)
	2015	67 (10.5)
	2016	69 (10.8)
	2017	112 (17.5)
	2018	102 (15.9)
	2019	106 (16.6)
	2020	107 (16.7)
	2021	64 (10.0)

### Characteristics of the childhood TB patients

Six hundred and seventeen (96.4%) of the children were new TB patients. Three hundred and sixty-seven (57.3%) of the children had EPTB, while the remaining 273 (42.7%) had PTB; 159 (24.8%) pulmonary-negative and 114 (17.8) pulmonary-positive. Among the EPTB patients, 210 (57.3%) had TB lymphadenitis, 26 had pleural TB (7.1%), 25 had osteoarticular TB (6.8%), 24 had intestinal TB (6.5%), and 22 had TB meningitis (6.0%).

Of 273 PTB patients, 120 (44.0%) were diagnosed using GeneXpert MTB/RIF®, 196 (71.8%) using a sputum smear microscopy test, and 43 (15.8%) using both tests. One hundred thirteen (20.8%) of all TB patients were examined with GeneXpert, and Mycobacterium tuberculosis (MTB) was found in 84 (63.2%); 79 (59.4%) had rifampicin resistance (RR) not detected, and 5 (3.8%) had RR indeterminate. MTB was not detected in the remaining 49 (36.8%) patients who underwent GeneXpert testing.

Thirteen (9.8%) of the 133 GeneXpert tests were performed on non-sputum samples such as lymphoid discharge, cerebrospinal, pleural or peritoneal fluids.

Of 196 PTB patients, 67 (34.5%) were positive for sputum smear microscopy test.

One hundred and ninety-four children (30.3%) children were undernourished when they began TB treatment; 109 (17.0%) had MAM and 85 (13.3%) had SAM. Only 13 (6.7%) of undernourished children received nutritional assistance (plump nut or plump sup). One hundred and sixteen (18.4%) of the 629 children who survived for two or more months after starting TB treatment were undernourished: 77 (12.2%) were MAM and 39 (6.2%) were SAM.

During the intensive phase of the treatment, 18 children (2.8%), not shown in the table, had at least one dose missed.

All 640 children had known HIV status, and 85 (13.3%) were living with HIV.

The BCG vaccination status was known for 524 (81.9%) of the children, and it was discovered that 315 (60.1%) had received the vaccine within two weeks of birth.

Of the 524 children, 56 (10.7%) were leaving with a cigarette-smoking family member.

Of the 640 children, 557 (87.0%) had not had a prior or concurrently known sick family member with TB living with them, while 78 (12.2%) had had at least one known PTB sick household contact; 77 (12%) had smear positive contacts and 1(0.2%) smear negative contact. Only 16 (20%) of the 80 children under the age of two had had PTB contact in the home (Table 3).

**Table 3:** Miscellaneous characteristics of childhood TB patients (≤16 years old) in central Ethiopia, 2014–2022

Characteristics	Category	Frequency (%)
TB treatment history (*n* = 640)	New	617 (96.4)
	Relapse	23 (3.6)
TB type (*n* = 640)	Extra-pulmonary	367 (57.3)
	Pulmonary-negative	159 (24.8)
	Pulmonary-positive	114 (17.8)
Extra-pulmonary TB type (*n* = 367)	TB lymphadenitis	210 (57.2)
	Pleural TB	26 (7.1)
	TB of the spine or bone	25 (6.8)
	Intestinal TB	24 (6.5)
	TB meningitis (CNS TB)	22 (6.0)
	TB peritonitis	12 (3.3)
	Other	1 (0.3)
	Not recorded	47 (12.8)
GeneXpert MTB/RIF® result (*n* = 133 )	MBT detected, RR not detect	79 (59.4)
	MTB detected, RR indeterminate	5 (3.8)
	MBT not detected	49 (36.8)
Sputum smear result (*n* = 194)	Positive	67 (34.5)
	Negative	127 (65.5)
Child’s nutritional status at the time of TB treatment start (*n* = 640)	Normal	446 (69.7)
	MAM	109 (17.0)
	SAM	85 (13.3)
Child received nutritional support (*n* = 194)	Yes (plump nut or plump sup)	13 (6.7)
	No	181(93.3)
Child’s nutritional status if survived to two months after starting TB treatment (*n* = 629)	Normal	513 (81.6)
	MAM	77 (12.2)
	SAM	39 (6.2)
Child’s HIV status (*n* = 640)	Positive	85 (13.3)
	Negative	555 (86.7)
BCG vaccination at birth or within 15 days of birth (*n* = 524)	Received	315 (60.1)
	Not received	209 (39.9)
A smoking family member living with the child (*n* = 524)	Yes	56 (10.7)
	No	468 (89.3)
A previous or concurrent household TB contact (*n* = 640) in child’s age	No household TB contact	557 (87.0)
	Pulmonary-positive	77 (12.0)
	Pulmonary-negative	1 (0.2)
	EPTB	5 (0.8)
Prior or concurrent TB type in household in a child under the age of two (*n* = 80)	Pulmonary-positive	16 (20.0)
	Pulmonary-negative	0 (0.0)
	EPTB	2 (2.5)

### Childhood TB disaggregated by age

Eighty (12.5%) of TB-sick children were under the age of two, while 157 (24.5%), 94 (14.7%), 205 (32%) and 184 (28.8) were under the age of five, between the ages of five and nine, ten to fourteen and fifteen to sixteen years old, respectively.

Children under the age of two made up 25.0% of all deaths. Children aged 0–4, 5–9, 10–14, and 15–16 years accounted for 41.7%, 22.2%, 25.0%, and 11.1% of all deaths, respectively. Of the 640 children who began TB treatment, 36 (5.6%; 95% CI = 4.0–7.7%) died during the treatment (Table 4). There were five (0.8%) lost-to-follow-up patients recorded on TB registers, but we confirmed their deaths through phone interviews with their caregivers and thus analyzed them as dead outcome. Except for one death, all of the deaths happened within 6 months of starting TB treatment. The remaining 604 patients (94.4%; 95% CI, 92.3–96.0%) were censored (i.e., cured, treatment completed, or treatment failed).

**Table 4:** The number of patients and deaths by age group among TB-sick children receiving TB treatment in central Ethiopia, 2014–2022

Age (years)	Number of TB patients (%), *n* = 640	Deaths (%), *n* = 36
Under 2	80 (12.5)	9 (25.0)
0–4	157 (24.5)	15 (41.7)
5–9	94 (14.7)	8 (22.2)
10–14	205 (32.0)	9 (25.0)
15–16	184 (28.8)	4 (11.1)
Over all death rate = 36/640 (5.6%; 95% CI = 4.0–7.7%)		

The following line graph depicts the age-related trend of TB disease frequency and the proportion of TB deaths shared by each age from the total TB deaths (i.e., 36). After declining after the age of two, the number of TB patients began to trend upward again around the age of 12 and peaked at the age of 16.

**Fig. 1:**
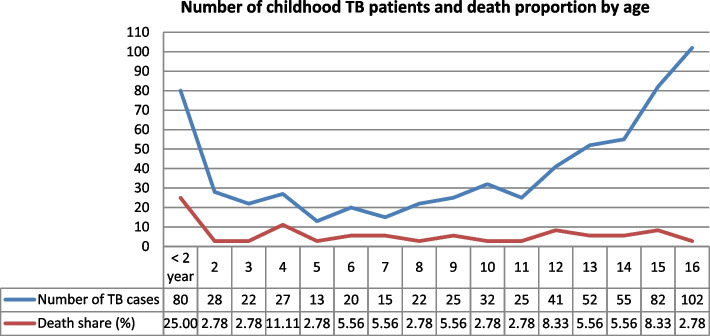
The distribution of childhood TB patients and death rates by age in central Ethiopia, 2014–2022

When we looked at the death share in percentage of total death by age for children who died while on TB treatment, we noticed that children under two years old had the highest share (25%), and the death share seemed stable, remaining below 10% for practically all ages equal to and beyond two years (Fig. 1).

### Predictors of death among children on TB treatment

The log-log plot satisfied PH assumptions for sex, nutritional status at the start of TB treatment, nutritional status two months later, age category, TB treatment history, and an interaction term of BCG vaccination with age category. The graphical assumption test for HIV status, on the other hand, showed diverging curves, indicating that PH was ambiguous. However, all of the covariates, including HIV status, passed the PH assumption test with p-value greater than 0.05 on the goodness-of-fit test. We fit both the standard Cox PH models and the extended Cox PH to resolve the discrepancy between the graphical and statistical assumption tests for HIV status. Ultimately, we chose Cox PH as the best-fit model because there was no significant difference in the two models’ outputs based on the likelihood ratio (LR) test statistics for model selection (likelihood ratio chi2 [1] = 0.93; *p*-value = 0.33) but the PH Cox model was more precise. Aside from the LR test and precision, the fitted extended Cox model revealed that the Cox regression coefficient estimate for the time-varying covariate, HIV status times log of survival time, was not statistically significant to be preferred (α = -0.72; 95% UI = -2.25–0.80; *p*-value = 0.35), implying that the risk of death did not differ significantly over time between HIV positive and HIV negative children receiving TB treatment.

In the multivariable model, HIV positive children were four times more likely than HIV negative children to die from TB (aHR = 4.21; 95% CI = 1.90–9.32). Similarly, undernourished children at the start of TB treatment were four times more likely to die than their normally nourished counterparts (aHR = 4.21; 95% CI = 2.21–10.48). Children under the age of ten were four times as likely as those aged ten and up to die (aHR = 4.06; 95% CI = 1.70–9.67). Children with relapsed or retreated TB were also more than three times more likely to die from the disease than children with new TB (aHR = 3.71; 95% CI = 1.05–13.11). There was no statistically significant effect modification (interaction) found between BCG vaccination and age categories labeled as under ten years and ten years or older (aHR = 0.76, 95% CI = 0.28–2.08) (Table 5).

**Table 5:** Cox proportional hazards survival analysis with single and multivariable covariates in children treated for TB in central Ethiopia, 2014–2022

Characteristics	Deaths/n (%)	HR (95% CI)	*p*-value	aHR (95% CI)	*p*-value
**Sex**					
Male	20/272 (7.35)	1.00		1.00	
Female	16/368 (4.35)	0.58 (0.30–1.13)	0.110	0.78 (0.37–1.64)	0.506
**Child’s HIV status**					
Negative	23/555 (4.14)	1.00		1.00	
Positive	13/85 (15.30)	3.98 (2.02–7.87)	0.000	4.21 (1.90–9.32)	0.000^*^
**Nutritional status at TB treatment start**					
Normal	15/446 (3.36)	1.00		1.00	
Undernourished (MAM or SAM)	21/194 (10.82)	3.31 (1.70–6.42)	0.000	4.81 (2.21–10.48)	0.000^*^
**Age category**					
≥ 10 years	13/389 (3.34)	1.00		1.00	
< 10 years	23/251 (9.16)	2.74 (1.39–5.42)	0.004	4.06 (1.70–9.67)	0.002^*^
**TB treatment history**					
New	33/617 (5.35)	1.00		1.00	
Relapse (retreatment after lost to follow up)	3/23 (13.04)	2.59 (0.79–8.47)	0.115	3.71 (1.05–13.11)	0.041^*^
BCG*age category					
Aged ≥10 and BCG vaccinated	23/464 (4.96)	1.00		1.00	
Aged < 10 or BCG unvaccinated or both	6/60 (10.00)	2.17 (0.88–5.36)	0.092	0.76 (0.28–2.08)	0.590

^*^Statistically significant at *p*-value < 0.05

**Fig. 2:**
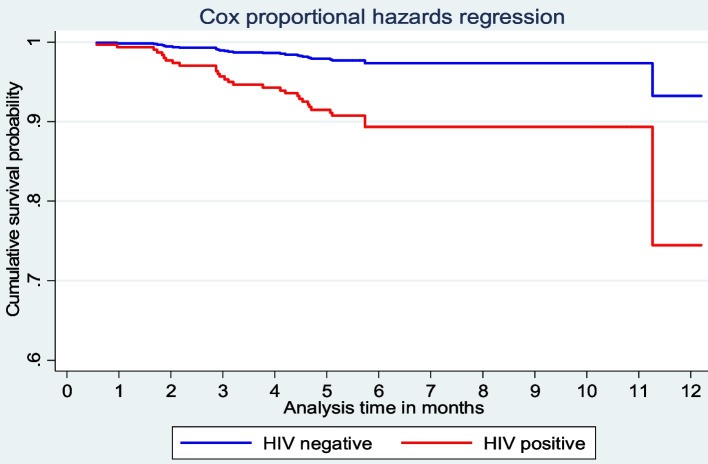
Estimated survivor function in HIV-negative and HIV-positive children receiving TB treatment

**Fig. 3:**
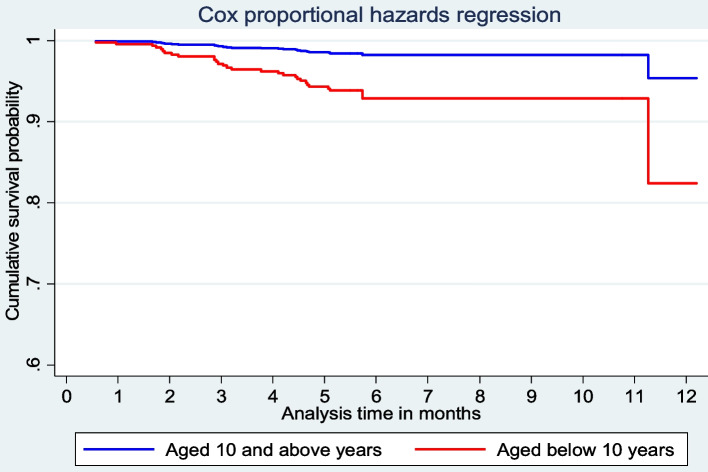
Estimated survivor function for children under the age of ten and those ten and older treated for TB disease

The post-Cox regression survival curve estimation revealed that the survival curve for the HIV negative children ran consistently above the curve for the HIV positive children over the course of time, indicating that the HIV negative group had a better survival probability (Fig. 2). Similarly, the survival curve for children aged ten and up remained higher than that of children under ten, indicating that older children outlive younger ones (Fig. 3). In a different multivariable Cox PH model (adjusting for gender, HIV status, age category, and TB treatment history), undernourished children were found to be significantly more likely to die than normally nourished children two months after starting TB treatment (aHR = 5.64, 95% CI = 2.42–13.14), not shown in a table.

The effects of BCG vaccination clearly violated the graphical assumption test by displaying crossing log-log curves at around survival time of three months. The time period disaggregated analyses for either time period, i.e., before and after three months, demonstrated no significant difference in survival between vaccinated and unvaccinated children; the HR for unvaccinated children starting TB treatment was 0.42 (95% CI = 0.13 − 1.36) in the first three months after treatment initiation and 1.22 (95% CI = 0.46 − 3.29) after the first three months after treatment initiation.

EPTB, smear-negative TB, and smear-positive TB were responsible for 18 (50.0%), 15 (41.7%), and three (8.3%) of the deaths, respectively. Meningitis TB claimed five lives, accounting for 27.8% of EPTB-related deaths and 13.9% of all TB deaths.

## Discussion

This study describes epidemiology of childhood TB and reveals predictors of death in children who received TB treatment in central Ethiopia between 2014 and 2022. Our four main findings are that; healthcare facilities in central Ethiopia had a high child TB death rate (5.6%), with children under two years old disproportionately affected; HIV, under nutrition at TB treatment initiation, persistent under nutrition throughout initiation phase, relapse, and young age all influenced mortality; BCG vaccination status at birth or within two weeks after birth did not influence mortality; and community transmission may be more important than household transmission among children of all ages.

The line graph in our result showed that the proportion of TB deaths were higher in the first few years of life and this is consistent with the literature [36]. This could be because infants’ immune systems are immature, making them more likely to die from TB irrespective of BCG vaccination [37].

Our study’s death rate (5.6%; 95% CI = 4.0–7.7%) is significantly higher than that of an earlier study in eastern Ethiopia (1.0%; 95% CI = 0.6–1.5) [21]. The eastern Ethiopia study did involve TB meningitis patients, which may explain the lower death rate observed in its findings, as TB meningitis has a 20% case fatality rate [38]. TB Meningitis accounted for nearly 14% of all TB deaths in our study.

When we compare our study’s death rate to the death rates of other African studies, we find that South Africa (0.8%; 95% CI = 0.7–0.9%) and the Democratic Republic of the Congo (1.4%; 0.4–3.6) appear to have significantly lower death rates. Lower death rates in both studies, however, were likely to be understated due to larger proportion of lost to follow-up, 7% in Congo [39] and 6% in South Africa [11], which could be due to deaths. In our study, however, patients recorded as lost to follow-up on the programmatic TB register were confirmed dead by phone call from their caregivers.

The death rate in our study is significantly lower than in a study conducted in rural southern Mozambique (10.7%; 95% CI = 8.7 − 12.8). One possible explanation for the higher death rate in the Mozambique study is that the majority of the patients (62%) were HIV co-infected, and nearly half (49.6%) were under the age of five [19], compared to a smaller proportion of patients being HIV co-infected (13.3%) and were under the age of five (24.5%) in our study. People who are TB-HIV co-infected have a higher risk of death than those who are not HIV infected, even if they are on anti-retroviral therapy, and younger children also have a higher risk of death than their older counterparts [10].

Other African countries’ death rates, Kenya (4%; 95% UI = 4.11–4.63%) and Malawi (9.5%; 95% CI = 6.4–13.4%), are comparable to this study’s findings, despite having higher TB-HIV co-infection rates, 28.0% for Kenya and 32.6% for Malawi. On the other hand, TB meningitis rates were lower in both the Kenya and Malawi studies, at 1% and 1.4% of all TB patients, respectively [18, 20]. A Nigerian study also discovered a comparable death rate (6.0%; 95% = 4.2–8.4%) despite a higher HIV co-infection rate (26.7%). However, it appears that Nigeria’s death rate was understated, as the proportion of patients that were lost to follow-up (possibly due to deaths) was high (15.0%) [23].

We discovered HIV to be an independent predictor of death, which is consistent with a recent meta-analysis finding [10].

Similar to our findings, Hesseling, et al., but it was limited to HIV-positive children, reported in South Africa that under nutrition at the time of diagnosis predicts mortality. The study did not, however, assess the effect of nutritional status after two months of treatment, which remained a significant predictor in our study [40].

Consistent with our findings, studies in Sidama Zone, Ethiopia [24], rural southern Ethiopia [9], Kenya [18], South Africa [11], and Nigeria [23] found that children of a younger age were at a higher risk of death, whereas studies in Malawi [39], and an older study in South Africa by Hesseling, et al. [40] did not. In contrast to our findings, re-treatment was not related to death in studies conducted in South Africa and Mozambique [11, 19].

The fact that only one in eight (12.2%) of all children and only 20% of those children < 2 years had a known previous or concurrent household PTB patient contact suggests that the children were infected with TB from outside sources. This is consistent with the findings of Martinez, et al. [41]. School community screening is not part of Ethiopia’s TB contact tracing and screening strategy, but is where children spend a significant amount of time [28].

In this study, the percentage of under nutrition among children under the age of five (21.7%, not shown in a table) at the start of TB treatment was higher than that of the percentage of under nutrition among the population of under the age of five in Ethiopia in 2016 and 2019, which were 10% and 7%, respectively [42, 43]. The rate of under nutrition two months after starting TB treatment in children under five (12.6%, not shown in a table) is still higher than the national figures. A gap in childhood TB research is revealed by the lack of studies on the deaths of children on TB treatment and their association with malnutrition on the African continent, where malnutrition is the highest in the world [44].

We also assessed the impact of the BCG vaccine on TB-associated mortality. Although BCG had no effect on mortality in our study, there is strong evidence that it prevents TB associated deaths [15, 45]. The difference could be due to the fact that the family of a deceased child may have falsely reported that the child had been vaccinated, resulting in a biased result on the effect of BCG in our study. The WHO recommends that all healthy newborns receive a single dose of BCG vaccine at birth or as soon as possible after birth to protect the child before infection occurs [46]. The Ethiopian Expanded Program on Immunization (EPI), which began in 1980, also calls for BCG vaccination to be administered at birth. If BCG is not given at birth, it can be given to children under the age of one year [47]. BCG coverage in Addis Ababa was 97.5, 94.6, and 96.3% in 2011, 2016, and 2019, respectively, while it was 81.6, 88.8, and 88.8% in urban Ethiopia in the same order [42, 43, 48]. To avoid the effect of prior infection before vaccination, which masks the effect of the vaccine, we assessed BCG vaccination status at birth or within 15 days after birth [49].

The line graph in our result showed a relatively low number of TB patients treated between the ages of two and 11 years, followed by a significant increase at later ages. This could be attributed to the impact of the BCG vaccine, which is effective in preventing TB for up to ten years and then fades [50]. However, infants’ immune systems are immature, making them more likely to develop TB disease regardless of BCG vaccination [37].

### Strengths and limitations

As strength of our study, supplementing the TB register review with a phone interview allowed us to capture data not available on the registers as well as confirm some data on the register. If we had not screened by phone and excluded those who died from misdiagnosed causes, the number of TB deaths considered for this analysis would have been 40 out of 650 (6.2%; 95% CI = 4.4–8.3%) instead of 36 out of 640 (5.6%; 95% CI = 4.0–7.7%). If the misdiagnosis proportion of 1.9% of those we interviewed by phone was applied to the 116 we could not reach by phone, there would be only two children who could have been misdiagnosed, and that would have a negligible effect on our results. The other strength of our study is that, unlike other studies in Africa, we have assessed the effect of persisted under nutrition to two months after TB treatment start on mortality and that of BCG.

The limitations of this study include the inability to determine the amount of milliary TB. This was due to the fact that these data are not collected in the TB register book and could not be confirmed with caregivers due to its medical and complex nature.

The fact that BCG vaccination status was determined through a phone call and caregivers of deceased children may have been influenced by social desirability bias to report a positive response that the child had been vaccinated, was likely to obscure the true effect of the BCG vaccine on mortality from TB.

Another limitation stems from a secondary data we used, which means that TB risk factors like diabetes were not recorded, and this study was unable to measure blood sugar in children and assess the association.

## Conclusions

The fact that the majority of TB-sick children spent the majority of their day in a crowded setting, such as school or daycare, and that only one in eight children had a previously or concurrently TB-sick family member with pulmonary TB suggests that schools, where children spent the majority of their day, may be a hotspot for TB transmission in children. Therefore, Ethiopia’s TB contact tracing and screening strategy may need to include close contacts including those in the child’s school community, where they spend a significant amount of time indoors.

The death rate among children on TB treatment was unacceptably high, affecting children under the age of two disproportionately. Focused intervention such as prevention of HIV infection, improving nutritional status of children on TB treatment, special attention for younger age children with TB, and prevention of recurrent TB should be implemented to minimize death among children on TB treatment.

The finding of BCG had no significant effect on survival should be interpreted with caution because it is likely to be prone to a potential social desirability bias of reporting positive responses on BCG vaccination by the parents in fear of a blame for children who died.

## Data Availability

The datasets used and/or analyzed during the current study are available from the corresponding author on reasonable request.

## Abbreviations

aHR: adjusted hazard ratio
BCG: Bacillus Calmette Guerin
BMI: body mass index
CI: confidence interval
CNS: central nervous system
EPTB: extra-pulmonary tuberculosis
HIV: human immune-deficiency virus
HR: hazard ratio
LR: likelihood ratio
MAM: Moderate acute malnutrition
MTB: Mycobacterium tuberculosis
MUAC: mid-upper arm circumference
PH: proportional hazards
PTB: pulmonary tuberculosis
RR: rifampicin resistance
SAM: severe acute malnutrition
SPH: school of public health
TB: tuberculosis
WH/L: weight for height/length
WHO: world health organization
